# Overall assessment system of combined mammography and ultrasound for breast cancer screening in Japan

**DOI:** 10.1007/s12282-020-01203-y

**Published:** 2021-01-02

**Authors:** Koji Ohnuki, Eriko Tohno, Hiroko Tsunoda, Takayoshi Uematsu, Yasuo Nakajima

**Affiliations:** 1 Overall Assessment Committee of the Japan Association of Breast Cancer Screening, Division of Breast and Endocrine Surgery, Iwate Prefectural Central Hospital, 1-4-1 Ueda, Morioka, Iwate 020-0066 Japan; 2 Study Group for Breast Cancer Screening in Japan Association of Breast and Thyroid Sonology, Iwate Prefectural Central Hospital, 1-4-1 Ueda, Morioka, Iwate 020-0066 Japan

**Keywords:** Breast cancer screening, Mammography, Ultrasound, Dense breast, Overall assessment

## Abstract

Mammography is the only screening method with evidence in support of reduced breast cancer mortality, but yields poor accuracy outcomes in women with dense breast tissue. The Japan Strategic Anti-cancer Randomized Trial (J-START) was conducted as part of the measures implemented to deal with women with dense breast tissue. Although the sensitivity was increased, the specificity was significantly lower in the intervention group because further examination was required in all positive cases classified by either mammography or ultrasound in the J-START. To address the issue, an overall assessment system of combined mammography and adjunctive ultrasound for breast cancer screening has been developed in Japan. The basic concept is based on a comprehensive assessment that includes a mammography assessment followed by an adjunctive assessment for ultrasound similar to the clinical setting. Currently, mammography alone is recommended for population-based breast cancer screening in Japan, but additional ultrasonography is extensively available for women, especially for women with dense breasts with shared decision-making for personalized breast cancer screening. The overall assessment system is recommended for use in Japan when breast cancer screening is conducted using both mammography and ultrasonography. In this article, we summarize the advantages of the overall assessment and the simultaneous mammography/ultrasound method, the basic approach used in Japan to assign the overall assessment category for breast cancer screening, and we outline the future directions of adjunctive screening ultrasound.

## Introduction

Mammography is the only breast cancer screening method used that has provided evidence in support of reduced breast cancer mortality. Nevertheless, it has been associated with a poor accuracy in the cases of women with dense breast tissues. In Europe and the United States, the age-specific incidence of female breast cancer peaks at age 60 and above. However, in Japan, it peaks at ages 40–49. In addition, similar to other countries, the proportion of women with dense breast tissues is high in the 40-year-old age group. Therefore, Japanese women with ages between 40 and 49 years are affected by the high-breast cancer incidence and the much denser breast tissue, thus resulting in lower mammography sensitivity. Thus, measures are needed for denser breast tissues. Nevertheless, it is a very tough challenge to conduct breast cancer screening in Japan.

Since 2007, the Japan Strategic Anti-cancer Randomized Trial (J-START), a randomized controlled trial that incorporated ultrasound in the screening mammography exam for women in the 40s, has been initiated in Japan as part of enforced measures to deal with women with dense breasts [1, 2]. Preliminary results from the J-START showed (a) that the sensitivity was significantly higher in the intervention group compared with the control group, and (b) that there was a significant reduction in interval cancers in the intervention group [3]. This explains why a breast cancer mortality reduction is expected, but this has not been proven yet. Conversely, the specificity was significantly lower in the intervention group because additional examinations are required in all positive cases classified in the J-START by either mammography or ultrasound. The assessment always results in low specificity when ultrasound is added to the screening mammography exam because the sensitivity and specificity in test performance always have trade-off relation for average-risk women. To address this issue, an overall assessment system of the combined mammography and adjunctive ultrasound for breast cancer screening was developed. In 2012, the Japan Association of Breast and Thyroid Sonology formulated the criteria for the overall assessment [4], and in 2015, the Japan Association of Breast Cancer Screening (JABCS) published the manual for the overall assessment [5]. The basic concept of the overall assessment is based on the conduct of a mammogram assessment first, followed by an adjunctive ultrasound assessment, and a comprehensive assessment based on both types of exams, similar to the clinical setting.

Currently, mammography alone is recommended for population-based breast cancer screening in Japan, but additional ultrasonography is extensively available for women, and especially in women with dense breasts, based on shared decision-making. Therefore, the overall assessment system is recommended by the JABCS when breast cancer screening is performed using both mammography and ultrasonography. The purpose of this study is to introduce Japan's assessment system of combined mammography and ultrasound for breast cancer screening to the rest of the world.

Two types for the overall assessment system of combined mammography and ultrasound for breast cancer screening: simultaneous mammography/ultrasound method and separate mammography/ultrasound method.

If a screening mammography shows findings that require an additional examination, the subsequent step would involves an ultrasound exam. However, in many cases, the ultrasound shows only typical benign lesions or no abnormal findings, and further examinations become unnecessary. For example, in the cases of focal density that require an additional examination following screening mammography, targeted ultrasound determined that 43–70% of the cases were normal or typically benign [6–8]. A system in which mammography and ultrasound are performed at the same time, and in cases in which additional examinations are performed if either the mammography or ultrasound findings are positive, does not reduce the number of false-positive cases attributed to mammography alone, even though the ultrasound findings are available at the same time. The main purpose of the overall assessment is to reduce the harm of screening by determining that further examination are not necessary at the screening setting. In the results of combined screening of mammography and ultrasound in Japan, the recall rates of the overall assessment were 16–53% lower than those of the independent assessment [9–12].

When ultrasound is incorporated in a comprehensive screening mammography exam, we have two methods to assign the overall assessment. One method involves the execution of separate mammography and ultrasound exams in which an ultrasound is performed without the knowledge of the findings and assessment of the corresponding mammography exam. The method is called as separate mammography/ultrasound method. Another method is the simultaneous mammography/ultrasound method in which an ultrasound is performed based on the findings and the assessment of the corresponding mammography exam. Hand-held ultrasound is dependent on the skills of the examiner. It is more accurate to perform an ultrasound exam following the corresponding mammogram and refer to the findings of these exams. This approach provides a very common indication for breast ultrasound in the clinical setting. It is clear that ultrasound characterization of mammographic abnormalities is included in the evaluation and management of breast disease. The method is called as simultaneous mammography/ultrasound method.

Basic concepts and typical cases of the overall assessment category for combined mammography and ultrasound for breast cancer screening.

Mammography and ultrasound are evaluated according to their respective Japanese guidelines [13–15], and then the final category is then decided using the criteria for the overall assessment of combined mammography and ultrasound for breast cancer screening (see Table 1). The categories are defined below.

1. Category 1: Negative. No additional examination required.
2. Category 2: Benign. No additional examination required.
3. Category 3: Low likelihood of malignancy. Need an additional imaging evaluation.
4. Category 4: Moderate likelihood of malignancy. Biopsy should be considered.
5. Category 5: Highly suggestive of malignancy. Appropriate action is required.

**Table 1:** Summary of the criteria used for the overall assessment of combined mammography and ultrasound for breast cancer screening

Type	MG findings		The role of adjunctive US	Influence on screening accuracy
A	C1, C2	Fibroglandular Density	Final category is decided by US	Sensitivity ↑
		Fatty density	US may not be necessary	
B	Mass C3-5	Circumscribed margin (C3)	Final category is decided by US	Specificity↑
		Uncircumscribed margin (C4, 5)	Further examination is usually required	
C	Asymmetries (C3–4)		Final category is decided by US	Specificity↑
D	Calcifications (C3–5)		Further examination is usually required	
E	Architectural distortion (C4)		Further examination is usually required	

*MG* mammography, *US* ultrasound, *C1* Category 1 (negative), *C2* Category 2 (benign), *C3* Category 3 (low likelihood of malignancy), *C4* Category 4 (moderate likelihood of malignancy), *C5* Category 5 (highly suggestive of malignancy) C3 or higher need further examination

In 2019, the Japanese Breast Cancer Society defined the screening categories as (a) the screening mammography categories (SMCs) for mammography evaluations, (b) the screening ultrasound category (SUC) for ultrasonic evaluations, and (c) the screening category (SC) for final determination. Further examinations are necessary for SC 3 or higher, that has the same meaning as category 0 in ACR-BI-RADS.

Type A. If there is no malignant finding on mammography and there is a suspicious malignant finding on ultrasound.

- If the location has fibroglandular density on mammography, the final category is decided by the ultrasound findings.
- Mammography sensitivity is always high in fatty tissue, so if the lesion of concern on ultrasound is in the fatty area on the mammography, it is unlikely to be a lesion that requires further examination.

**Fig. 1:**
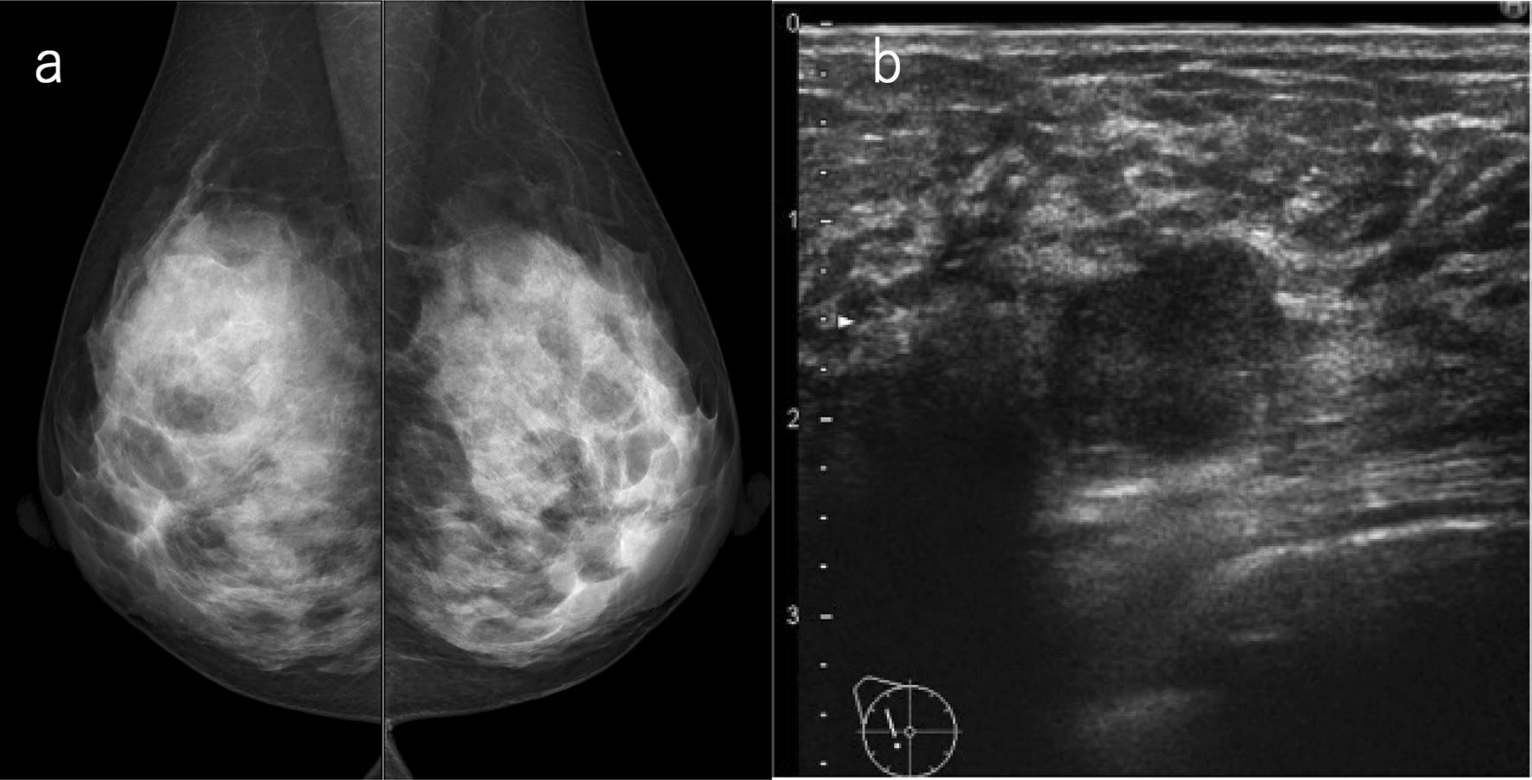
A case with no malignant findings on mammography. **a** Mammography: the breast composition is extremely dense, SMC 1. **b** Ultrasound: irregular, not parallel, hypoechoic mass, SUC 4. The overall assessment category: SC 4. Histopathology: invasive ductal carcinoma, grade 3 (*SMC* screening mammography category, *SUC* screening ultrasound category, *SC* screening category)

If mammography does not detect any malignancies (SMC1, 2), ultrasound is highly useful when the breast composition is heterogeneous or extremely dense. The ultrasound exam can detect occult cancers identified in mammography a priori, and can consequently increase sensitivity (Fig. 1). On the other hand, the fatty areas on the mammogram are not too much of a concern on ultrasound.

Lesions located in the periphery of the breast may not be projected on mammography. Even in the case in which breast composition is almost entirely fatty, or in cases with scattered areas of fibroglandular density, the classification of the final category is decided by the ultrasonic findings if the lesion detected by the ultrasound is considered to be present in the blind area of mammography.

Type B. If a mass of SMC 3 or higher is found on mammography.

- In the case of the circumscribed margin, the final category is decided by the ultrasonic findings.
- If the mass shows microlobulated, indistinct, or spiculated margins, the final category is decided by mammography.
- If the lesion corresponding to the mammography findings cannot be identified by ultrasound, the final category is decided by mammography.

**Fig. 2:**
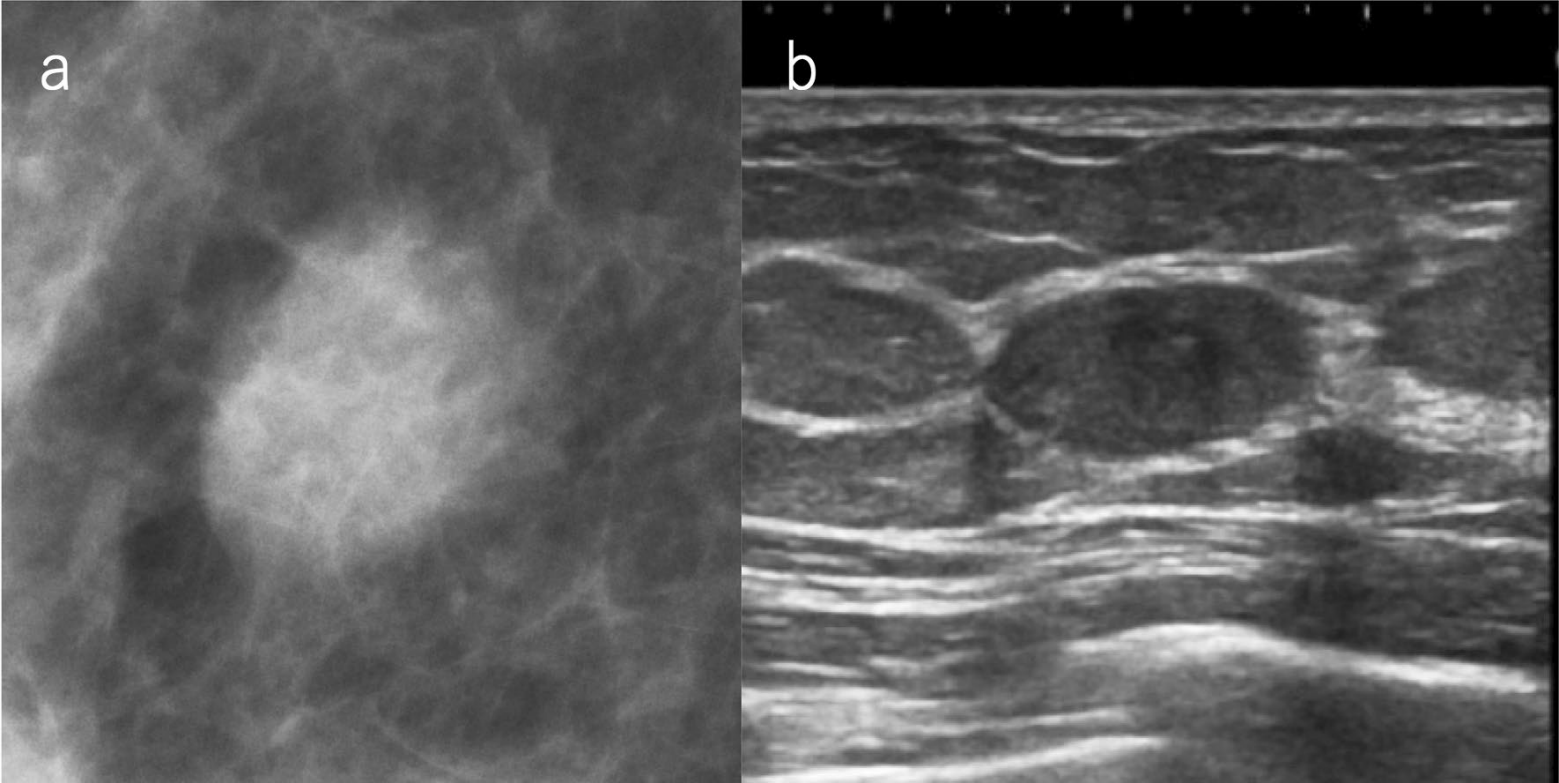
A case of circumscribed mass on mammography. **a** Mammography: oval mass with margin mostly obscured but also circumscribed, SMC 3. **b** Ultrasound: oval, circumscribed, and parallel to the skin; classically fibroadenoma, SUC 2. The overall assessment category: SC 2 (*SMC* screening mammography category, *SUC* screening ultrasound category, *SC* screening category)

Examples of lesions that are visualized as circumscribed masses on mammography include cysts, fibroadenomas, intracystic tumors, and breast cancers with minor tendency to invade surrounding tissues (mucinous carcinoma, etc.). Ultrasound is useful for the evaluation of the internal structure or orientation of the mass, and if there are findings of simple cysts or typical fibroadenomas, ultrasound findings can be prioritized and classified as SC 2 (Fig. 2). Even when a benign tumor, such as a cyst or fibroadenoma, is hidden by superimposed or adjacent fibroglandular tissue, and appears to have obscured margins, it can be classified as category 2 by ultrasound.

When mammography shows that the margins of the mass are microlobulated, indistinct, or spiculated, it is considered to reflect the invasion of breast cancer into surrounding tissues. If these findings are observed, the overall assessment should be performed based on malignancy.

**Fig. 3:**
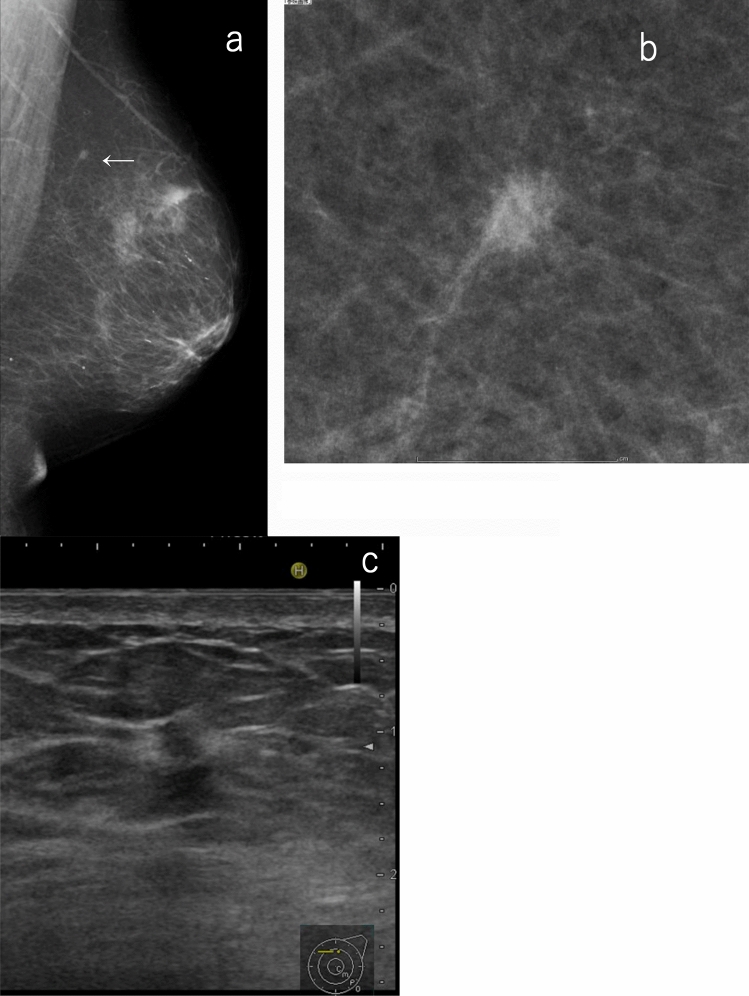
A case of a small, indistinct mass on mammography but no abnormal findings on ultrasound. **a**, **b** Mammography: small indistinct mass (allow), SMC. Screening ultrasound: no abnormality, SUC 1. The overall assessment category: SC 4. **c** Targeted ultrasound: indistinct mass with interruption of the anterior border of the mammary gland and halo sign (4 mm), DUC4. Histopathology: invasive ductal carcinoma. This case is difficult to detect based on screening ultrasound, especially when performing ultrasound without reference to mammography findings (separate mammography/ultrasound method) (*SMC* screening mammography category, *SUC* screening ultrasound category, *SC* screening category, *DUC* diagnostic ultrasound category)

It should be noted that a mass in the deep or the peripheral part of the large breast may not be easily detected by ultrasound. Further, a small invasive tumor existing in adipose tissue may not be easily detected by ultrasound even if it can be confirmed by mammography, especially when performing ultrasound without the findings and assessment of the corresponding mammography (separate mammography/ultrasound combined method). If there is a possibility (as described above), the mammography findings should be prioritized even if there are no abnormalities on ultrasound (Fig. 3). When ultrasound is performed based on the findings and assessment of the corresponding mammography (simultaneous mammography/ultrasound combined method), it is possible to perform targeted scanning on lesion candidates so that the ultrasound can be efficiently performed. In this sense, its reliability is enhanced.

Type C. If asymmetries are found on mammography.

- If asymmetries can be determined to be attributed to a normal mammary gland by ultrasound, this gland will be classified as SC 1.
- If the ultrasound can confirm a mass or non-mass abnormality, give priority to ultrasound. Attention should be paid to breast cancer with a predominant intraductal component and to non-mass abnormalities that appear as hypoechoic areas with indistinct margins.
- If the lesion corresponding to the mammography findings cannot be identified by ultrasound, the final category is decided by mammography.

**Fig. 4:**
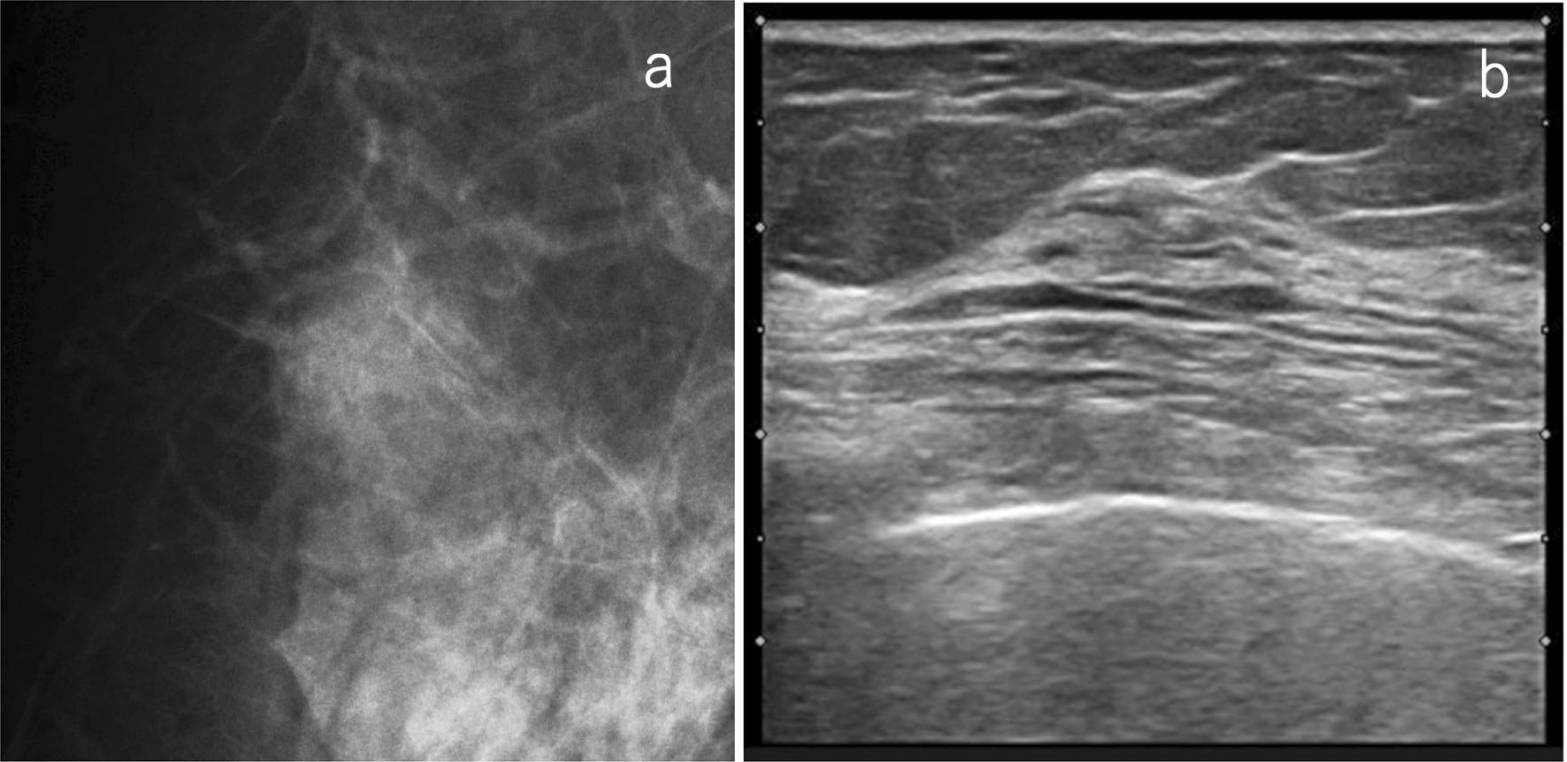
A case of focal density on mammography. **a** Mammography: focal density, SMC 3. **b** Ultrasound: focally thickened normal mammary glands were detected as abnormal findings on MG, SUC 1. The overall assessment category: SC 1 (*SMC* screening mammography category, *SUC* screening ultrasound category, *SC* screening category)

If the focal or global densities are found on mammography, it is necessary to estimate the location from two-view mammography and pay particular attention to the location during ultrasonic scanning. If there are findings in one view only, a larger area should be carefully scanned. In general, asymmetric densities on mammography are not likely to correspond to true pathological lesions. If the ultrasound is performed with special attention to the location, and there is no obvious abnormality other than the normal mammary tissue, the overall assessment will be category 1. This assessment avoids a number of additional, unnecessary examinations (Fig. 4). If the mammographic density of a certain size is attributed to a true lesion, the lesion is usually detected by ultrasound. If the ultrasound cannot reveal any lesion corresponding to that type of mammographic density and yields just a normal gland, this will constitute firm evidence in justification of category 1 classification.

Conversely, if the ultrasound shows a mass or non-mass abnormality at the location of the mammographic findings, the final category is decided by ultrasound.

**Fig. 5:**
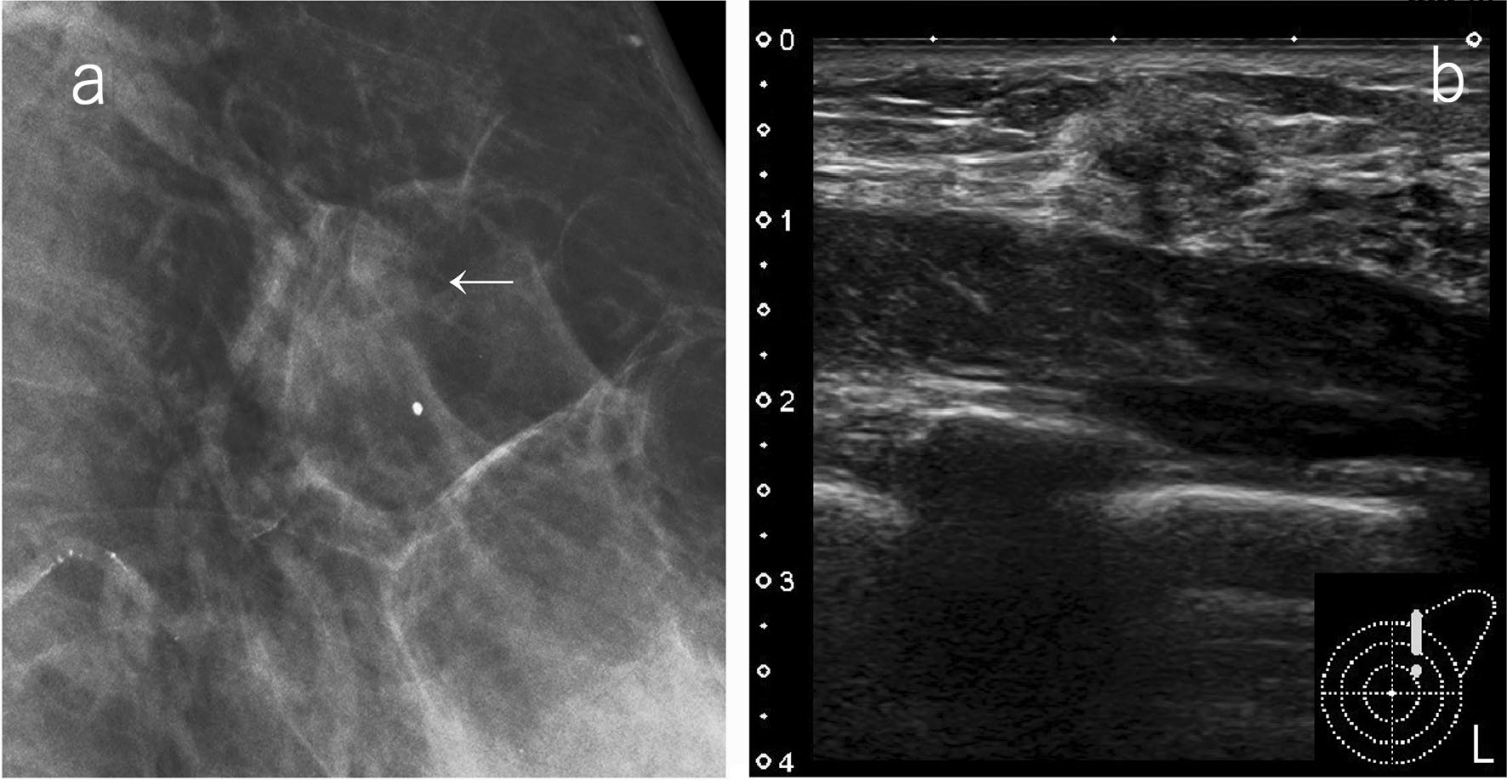
A case of focal density on mammography. **a** Mammography: focal density (allow), SMC 3 (SMC4 if it is developing asymmetry). **b** Screening ultrasound: hyperchoice mass with indistinct margin, SUC 4. Overall assessment category: SC 4. Histopathology: invasive ductal carcinoma. This case may be difficult to detect with screening ultrasound, especially when performing ultrasound without reference to mammography findings (separate mammography/ultrasound method) (*SMC* screening mammography category, *SUC* screening ultrasound category, *SC* screening category)

If the asymmetric density is small, and the internal echoes of the corresponding lesion are isoechoic to hyperechoic with fat, or if the asymmetric density is located just below the nipple, or at the margin of the mammary gland, it may be difficult to detect it by screening ultrasound. Specifically, in the case of the separate mammography/ultrasound method, if the lesion detected by mammography is considered difficult to detect by ultrasound, the mammographic findings should be prioritized (Fig. 5).

Type D. If calcifications of category 3 or higher are found on mammography.

- In principle, priority is given to mammographic findings. However, it may be possible to determine lesions as benign that are apparent by ultrasound.

If mammography shows calcifications of category 3 or higher, the findings of mammography should be given priority in principle. Regardless of the ultrasonic findings, further examination will be required. Therefore, it is not necessary to evaluate the calcifications themselves in detail by screening ultrasound, but it is necessary to carefully check the presence or absence of mass or non-mass lesion abnormalities in the area where calcifications are found in mammography. The final category may be more suspicious of malignancy than mammography if ultrasound detects a mass or non-mass lesion.

When the number of punctate calcifications is small and the density is low, the mammographic category is usually equal to two. However, when the background regions of calcifications have high densities, these calcifications may be the only findings that may indicate malignancy on mammography. Thus, it is more important to evaluate the presence or absence of mass or non-mass lesions at the corresponding site by ultrasound.

Conversely, even if there are category 3 calcifications in mammography, it may be possible to classify them as category 2 based on the overall assessment. For example, if mammography shows coarse heterogeneous calcifications but there is a tumor with a circumscribed margin, and the long axis of the lesion parallels the skin line at the site of calcification on ultrasound, the lesion is judged as a fibroadenoma and is classified as category 2.

Type E. If architectural distortion is found on mammography.

- In principle, the final category is decided by mammography.

**Fig. 6:**
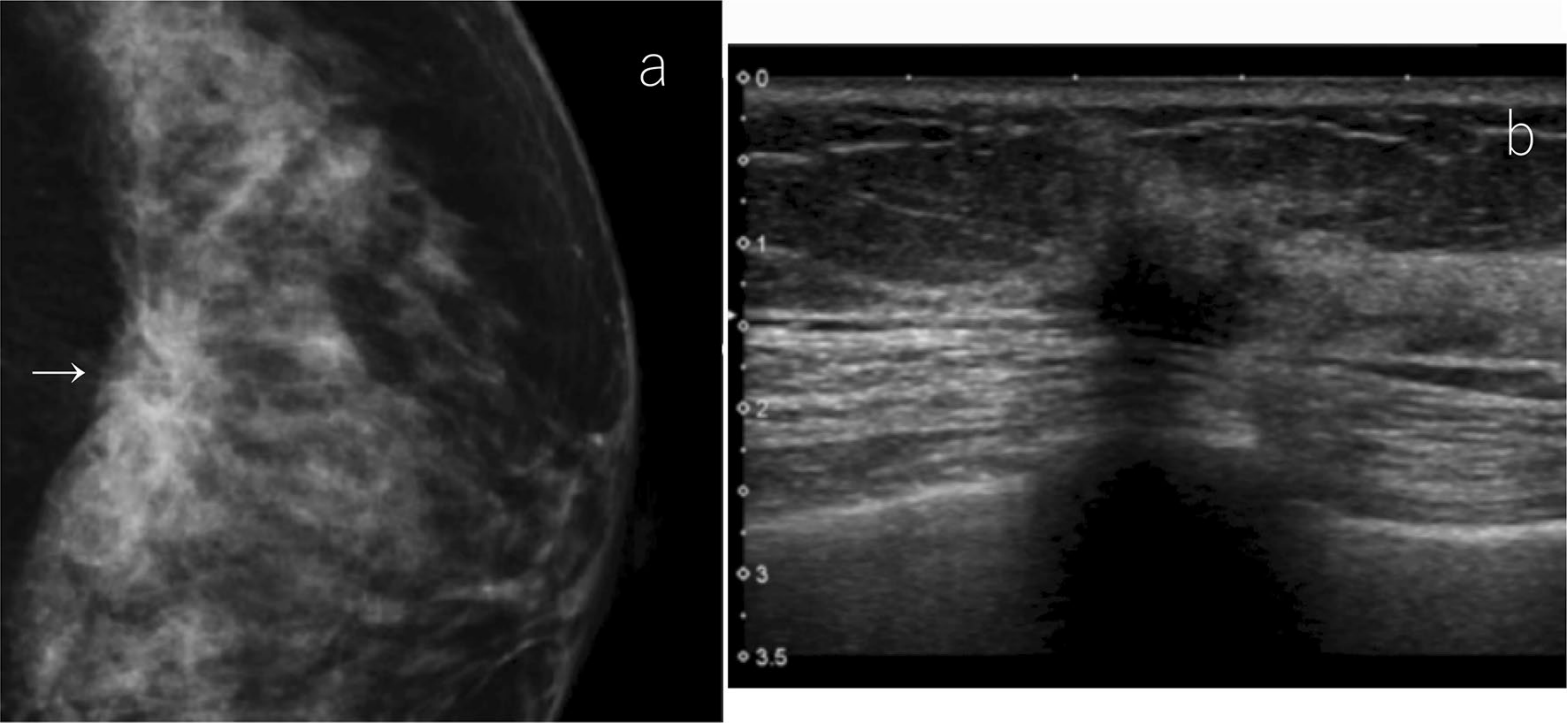
A case of architectural distortion on mammography. **a** Mammography: architectural distortion, SMC 4. **b** Ultrasound: irregular, not circumscribed, and not parallel to the skin with interruption of the anterior border of the mammary gland and halo sign. Typical findings of invasive cancer, SUC 5. The overall assessment category: SC 5. Histopathology: invasive ductal carcinoma (*SMC* screening mammography category, *SUC* screening ultrasound category, *SC* screening category)

If the mammography shows obvious architectural distortion and the person examined had no history of trauma or surgery, the finding is suspicious for malignancy. Given that it may be difficult to point out subtle architectural distortion during screening ultrasound, the mammography assessment should be prioritized even when there are no abnormal findings on ultrasound. If a mass with an echogenic halo is found on ultrasound at the site of the architectural distortion on mammography, the final category is classified as a category 5, even if the mammography classified it as a category 4 (Fig. 6).

## Practice of the overall assessment of combined mammography and ultrasound in breast cancer screening

Because breast ultrasound is almost mostly performed by the hand-held method in Japan, the accuracy of the ultrasound screening depends considerably on the examiners. Quality control of ultrasound is very important and is performed according to the quality assurance guidelines established by J-START [16].

When conducting a combined screening of mammography and ultrasonography, the JABCS recommends conducting the mammography exam first followed by the ultrasonic exam executed in reference to the mammography findings (simultaneous mammography/ultrasound method). Although it is ideal to perform an ultrasound with reference to the qualified physician's interpretation, in most cases in Japan, sonographers perform the ultrasound while they read the mammography results themselves. Owing to these circumstances, the Japanese Central Organization on Quality Assurance of Breast Cancer Screening (JCOQABCS) regularly holds mammography interpretation sessions for sonographers. Therefore, in Japan, sonographers can receive training for mammography interpretation in addition to training for ultrasound [17, 18].

To perform more accurate evaluations, it is better to record the entire breast ultrasound image either by volume data or video, blood flow, and/or elastography information of the detected lesion. These require time and cost. However, in ultrasound, as in mammography, unnecessary additional examination can be avoided by comparing the findings with previous images and movies. Communication between the sonographer and the physician is also important, and if there is a regular conference, the accuracy of the examination will increase.

The comprehensive evaluation is usually performed by one physician, in reference to the initial interpretation of the mammogram performed by another physician and the ultrasound records of the sonographer. The physician must have his/her qualifications certified by the JCOQABCS to conduct the overall assessment.

## Future directions of adjunctive ultrasound screening in Japan

When considering additional modalities of mammography screening, it is important to evaluate the benefits and harms. One of the harms to consider is a false-positive result. For dense breasts, the additional ultrasound to mammography screening has been reported to increase the recall rate by 5.5–15.1% [19, 20]. In the first screening round of J-START in the 40 s age group, the recall rate in the intervention group was limited to a 3.8% increase compared to the control group [3]. The Japanese recall criteria for ultrasound breast cancer screening are designed to avoid high recall rates [14, 15]. With learning curves and comparison to prior images, it is expected that the recall rate of the second screening round will decrease from the first screening in J-START. The additional recall rate of organized mammography screening for breast cancer combined with ultrasound on women aged 40–69 years in Austria [21] was 1.31%. In population-based screening, the recall rate could be kept lower than the prevalent screening. The overall assessment can reduce false positives even more than mammography alone when ultrasound is added to a mammography screening [22]. According to the results from several facilities in Japan, the overall assessment showed that the breast cancer detection rate was higher in dense breasts, and the recall rate was lower in non-dense breasts compared with mammography alone (unpublished data).

In addition, overdiagnosis must be considered as one of the harms, but is difficult to assess overdiagnosis at this time [23]. Ultrasound does not delineate punctate or amorphous calcifications without any other abnormality that is detected by mammography alone. This means that it does not detect low-grade DCIS and breast ultrasound may be less harmful than mammography in terms of overdiagnosis. However, whether small invasive cancers detected by ultrasonography alone are overdiagnosed is a subject for future study.

In addition, overdiagnosis must be considered as a harm, but is difficult to assess overdiagnosis at this time [23]. Ultrasound does not delineate punctate or amorphous calcifications without any other abnormality that detected by mammography alone. This means that it does not detect low-grade DCIS and ultrasound has an advantage for overdiagnosis compared with mammography. However, whether small invasive cancers detected by ultrasonography alone are overdiagnosed is a subject for future study.

Hand-held ultrasound devices have many advantages, such as their compactness (small sizes) and low costs, lack of exam pain, lack of radiation exposure, and the fact that they do not use contrast agents. However, given that ultrasonic exams are examiner dependent, there is a possibility for a shortage of trained technologists if ultrasound is extensively implemented in screening. Although more than 3000 breast ultrasound technologists have been certified by the JCOQABCS, many of them are engaging in breast and abdominal ultrasound exams. Therefore, the number of breast ultrasound technologists is still in shortage for population-based breast cancer screenings in Japan. In addition, spread of automated whole breast ultrasound system may be need.

Adjunctive ultrasound may be more useful for breasts with less adipose tissue that is common in the Japanese population. In addition, in Japan, screening of bilateral breasts with ultrasound costs 3500 yen with insurance, and it is considered cost effective. With adequate quality control and complementary use with mammography in the overall assessment, adjunct ultrasound may be not only constitute an alternative to magnetic resonance imaging exams for women with a high risk of breast cancer, but could also serve as an appropriate screening modality for women with an average risk of breast cancer. To determine whether ultrasound should be added to population-based breast cancer screening, proof of mortality reduction from long-term follow-up in J-START and appropriate cost-effectiveness analysis must be performed. Although the mortality rate reduction is the most important parameter used to evaluate the efficacy of adjunctive ultrasound in breast cancer screening, preliminary results from the J-START are essential in providing personalized supplemental screening modality choices to women with dense breasts.
